# Bioaerosol emissions associated with pit latrine emptying operations

**DOI:** 10.1016/j.scitotenv.2018.08.147

**Published:** 2019-01-15

**Authors:** Stewart Farling, Tate Rogers, Jackie S. Knee, Elizabeth A. Tilley, Joe Brown, Marc A. Deshusses

**Affiliations:** a Dept. of Civil and Environmental Engineering, Box 90287, Duke University, Durham, NC 27708-0287, USA; b Triangle Environmental Health Initiative, 711 N Hyde Park Ave, Durham, NC 27703, USA; c School of Civil and Environmental Engineering, Georgia Institute of Technology, 311 Ferst Drive, Atlanta, GA, 30332, USA; d Department of Environmental Health, University of Malawi, The Polytechnic, Chichiri, Blantyre 3, Malawi; e EAWAG: Swiss Federal Institute of Aquatic Science and Technology, Überlandstrasse 133, 8600 Dübendorf, Switzerland

**Keywords:** Bioaerosols, Sanitation, Pit latrines, Fecal pathogens

## Abstract

Pit latrines are the most common sanitation option in the developing world. They are simple to build but require periodic emptying which results in widespread dispersion of fecal pathogens in the environment. While much is known about the health risks of fecal-oral exposure, little is known about those resulting from the aerosolization of pathogens from fecal material. Bioaerosols were sampled around seven pit latrines before, after, and during emptying in Blantyre, Malawi. Bioaerosols were collected directly onto nutrient and selective medium agar plates using an impact sampler. DNA was extracted from some plates and analyzed for selected enteric pathogens. Total heterotrophic bacteria in the air during active emptying ranged from 198 to >13,000 colony forming units (CFU) per m^3^, and generally increased above background levels during pit emptying. At about one meter from the pit latrine emptying, *E. coli* and total coliforms concentrations in air reached up to 350 and 790 CFU m^−3^, respectively. Additionally, at four out of the seven pit latrines sites sampled, enterotoxigenic *E. coli* (ETEC) LT/ST was confirmed to be present in bioaerosols. This work demonstrates the potential for airborne dispersion of enteric pathogens during pit latrine emptying operations.

## Highlights

• Bioaerosols were sampled during pit latrines emptying in Blantyre, Malawi.
• *E. coli*, coliforms and enterotoxigenic *E. coli* (ETEC) were detected.
• High bioaerosols concentrations correlated with certain emptying practices
• The results highlight aerosolization of enteric pathogens during pit emptying.
• Further studies are needed to quantify exposure and health risks.

## Graphical abstract

**Figure:**
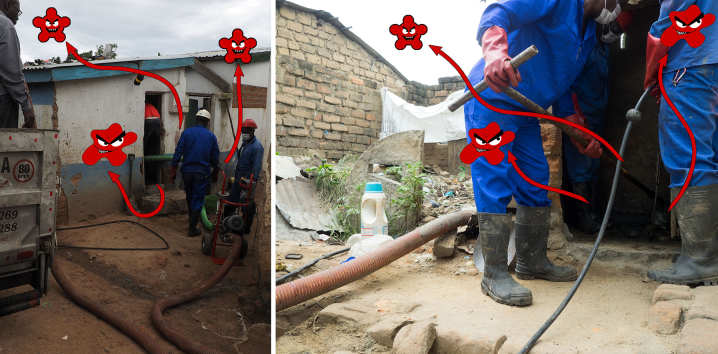

## 1 Introduction

Improving sanitation in the developing world has become a focus in recent decades, and is specified in the Millennium Development Goals (Organization, 2013) and now in the Sustainable Development Goals (UNDP, 2015). However, the World Health Organization estimates that around 525,000 children die each year due to diarrheal diseases, which are often directly related to lack of proper sanitation (World Health Organization, 2014). Safely managed improved sanitation has the potential to decrease child deaths and reduce morbidity.

Currently, on-site sanitation systems, such as pit latrines, are the most common sanitation option used throughout the developing world, and it is estimated that around 1.8 billion people use pit latrines as a main means of sanitation (Berendes et al., 2017; Graham and Polizzotto, 2013; Jenkins et al., 2015). Though simple to build and use, pit latrines and other onsite sanitation systems generally require periodic emptying, transport and treatment or disposal of the fecal sludge (Chunga et al., 2016; Mbéguéré et al., 2010; Sisco et al., 2015). Safe management of fecal sludge during the emptying process can be technically and logistically challenging. Even one of the more formal and seemingly hygienic methods of waste removal, operation of a vacuum tanker truck by trained personnel, may result in contamination of the surrounding environment and risk of exposure to fecal pathogens. It is generally accepted that the most common route of transmission for enteric pathogens is via the fecal-oral route (World Health Organization, 2004). Very little is known about the airborne exposure risks associated with mechanically agitating and pumping semi-liquid human feces in the developing world.

Bioaerosols are airborne matter that originates from microbes, plants, or animals (Yoo et al., 2017). Previous studies have found bioaerosols can originate from conventional flush-style toilets (Barker and Jones, 2005; Gerba et al., 1975; D. Johnson et al., 2013; D. L. Johnson et al., 2013; Verani et al., 2014), wastewater treatment plants and operations (Dowd et al., 2000; Heinonen-Tanski et al., 2009; Karra and Katsivela, 2007; Pepper et al., 2006; Sánchez-Monedero et al., 2008; Schlosser et al., 2011; Tanner et al., 2005; Uhrbrand et al., 2011), composting operations (Kummer and Thiel, 2008; Pahari et al., 2016; Sanchez-Monedero et al., 2003), and can pose risk of person-to-person transmission of pathogens in hospital settings (King et al., 2015, King et al., 2013; Verani et al., 2014). To our knowledge, no previous work has investigated the extent and content of bioaerosols generated during mechanized pit emptying practices. This work reports the results of one week of bioaerosol sampling performed during pit emptying activities in Blantyre, Malawi, which is a moderately dense, peri-urban informal settlement. Samples were analyzed to determine the concentration of specific bioaerosols and the presence of key pathogenic organisms.

## 2 Materials and methods

In December 2016, we shadowed a pit latrine emptying crew in Blantyre, Malawi. Our investigations were conducted in conjunction with a study of novel equipment designed to reject trash from pit latrines during emptying. Pit emptiers used a standard vacuum tank system to empty pit latrines using a prototype (Sisco et al., 2015) that included a trash rejecting system. The pits' contents were first “fluidized” by pumping water under the sludge surface with a pressure washer and then mixing by hand with a spiked rod. These are common practices used by pit emptiers. Once the sludge was deemed fluid enough for pumping, the trash rejecting hose was placed in the pit to begin sludge removal. If needed, the fluidization step was repeated. Any trash stuck to the spiked rod was removed by hand and set in a corner of the latrine.

### 2.1 Agar preparation

Nutrient and MI agars (Difco™) were prepared per manufacturer's instructions with the following field-based modifications due to lack of access to an autoclave: an electric tea kettle was used to boil distilled water for 5 min, then MI or nutrient agar powder was added directly to the kettle, mixed by hand using a metal spatula, and boiled for 5 min. The agar was cooled to 55–60 °C and poured into sterile plates. Plates from each batch were reserved for use as negative controls which showed absence of contamination.

### 2.2 Bioaerosol sampling

Air near target pit latrines was sampled using an MAS-100 ECO microbial air sampler (MBV AG, EMD Chemicals Inc. Gibbstown, NJ). The portable sampler relies on impaction directly onto agar plates and operates at a flowrate of 100 L/min. High volume (0.5–1.0 m^−3^ of air) and low volume (0.10–0.20 m^−3^ of air) samples were attempted before, during, and after emptying at 7 pit latrines. Temperatures were recorded during sampling and ranged from 30 to 33 °C. Wind direction was recorded on individual site drawings, but wind speed and relative humidity levels were not recorded. Non-specific nutrient agar was used to enumerate total heterotrophic bacteria and MI agar was used to enumerate *Escherichia coli* (*E. coli*) and total coliforms. DNA extraction was performed on bacterial growth recovered from agar plates. Not all combinations of sampling volumes and agars were taken at every pit due to logistical issues with the emptying procedures, stoppages, or emptying equipment adjustments occurring during sampling (see SI for listing of all samples taken). The locations of samples were selected to be as similar as possible between sites, but variations were required due to drastic differences between pit latrines design and location. The distance from the pit opening to the pit sample location therefore varied between 1 and 5 m. Background, pit emptying, pit cleaning, and post cleaning samples were taken at the same location during the operation. When pit emptying facilitated it, some locations could be sampled upwind and/or downwind as well. Samples related to the vacuum truck, such as the truck vent, were taken in the same location relative to the truck when possible. The location of the truck relative to the pit latrine varied from latrine to latrine depending on how the workers could position the vacuum truck near the latrine. In some cases, the truck was positioned behind a compound wall which physically separated the truck vent from the work space and workers (see site drawings and pictures in Supplementary Data). Locations with street access had the workers within sight of the truck. Post-emptying samples were at some pit latrines 30 to 60 min after emptying was completed. To measure passive deposit of bioaerosols on surrounding surfaces, nutrient and MI agar plates were set out in selected locations near the pit latrines. Locations were chosen based on an ad hoc basis depending on what nearby surfaces were available for placement of petri dishes (see SI – “Flat” samples). Locations chosen included a front porch, near a clothesline, and near an open kitchen window. See SI for a full list of sample locations and site drawings.

### 2.3 Bacterial culture methods

After sampling, agar plates were stored in a cooler without ice and transported to the lab within 8 h of collection. Plates were incubated at 35 °C overnight and colony forming units (CFUs) were counted the following morning. Since the ambient temperature after sampling and during transportation was close to the incubation temperature, total time for colony growth was 18 to 22 h. Final bacterial concentrations were calculated using the most probable number method, per instructions of the MAS-100 ECO sampler.

### 2.4 Sludge sampling

At 4 out of 7 of the pits (pits 2, 5, 6, 7), sludge samples were collected following fluidization. 1 mL of sludge was mixed with 100 mL of UV sterilized distilled water, and 2 mL of the resulting suspension was diluted 1:1 with UNEX buffer (Microbiologics, St. Cloud, MN), vortexed, and frozen for future molecular analysis. As will be discussed in the Results section, this turned out to be excessive dilution.

### 2.5 DNA extraction and molecular analysis

DNA was extracted from bacterial colonies that grew on the agar plates and from sludge samples. 42 of the aerosol samples showing good growth on agar were processed as follows: First, 2 mL of UV sterilized distilled water was filtered through a 0.2 μm filter directly onto the agar. A sterilized glass spreader was then used to gently rub the surface of the agar and detach the colonies. The resulting suspension was transferred to a SK38 glass bead tube (7 mL tubes (Precellys, VWR) with 2 mL of UNEX buffer (Hill et al., 2015) (Microbiologics, St. Cloud, MN). The SK38 tubes were vortexed for 10 min to lyse all cells. Diluted sludge samples (2 mL, see above) were processed in a similar fashion. The lysate was frozen and shipped to the Georgia Institute of Technology for analysis. 100 μL of samples were pretreated with stool ASL buffer (Qiagen, Germantown, MD, USA) per Luminex GPP protocol, and extracted using the QIAamp 96 Virus QIAcube HT kit (Qiagen, Germantown, MD, USA). We used the Luminex (Austin, TX, USA) Gastrointestinal Pathogen Panel (GPP), a multiplex end-point RT-PCR assay, for enteric pathogen detection. Targeted enteric pathogens included: *Campylobacter*, *Clostridium difficile* toxin A/B, *E. coli* O157, enterotoxigenic *E. coli* (ETEC) LT/ST, shiga-like toxin producing *E. coli* (STEC) stx1/stx2, *Salmonella*, *Shigella*, *Vibrio cholera, Yersinia enterocolitica.* The GPP assay also includes viruses and protozoa, which would not be detected in agar samples but could have been present in sludge samples. These included: *Cryptosporidium, Entamoeba histolytica, Giardia,* adenovirus 40/41, norovirus GI/GII, and rotavirus A.

## 3 Results & discussion

### 3.1 Environmental and sampling conditions

Conditions during sampling in Blantyre, Malawi were generally sunny, with light winds and temperatures from 30 to 33 °C. It was however the rainy season, which resulted in light rain on the first day of operations, and heavy rain during the emptying of pits 3 and 4, impacting our ability to collect air samples. Air sampling was a challenging exercise as pit emptiers were working rapidly and sampling for total coliforms and total heterotrophs was complicated by the fact only one air sampler was available, and sampling required 1–10 min per agar plate to achieve the target sample volumes at the fixed 100 L/ min air sampling rate. Future studies should consider the deployment of multiple samplers.

### 3.2 Quantification of total heterotrophic bacteria

Background (pre-emptying) total heterotrophic bacteria levels varied greatly from 135 to above a detection limit of 2628 CFU m^−3^. There was no obvious reason for the drastic variation in the background levels between sites based on any obvious characteristics at a given site, such as pit latrine design, or nearby trash pits. Pits 2 and 7 (high counts) were in home compounds with walls surrounding the entire complex, a setting very similar to pits 1 and 6 which had very low counts (see site drawings in Supplementary Data). At pit locations within detection limits (1,4,5 and 6), total heterotrophic bacteria increased once pit emptying began and decreased to near background levels 30 to 60 min after emptying operation ceased (Table 1). The rapid decrease of the bacteria levels after operations ceased would suggest that the most direct exposure risk may be to pit emptiers and by-standers, however the transport and deposition of such bioaerosols is not well understood. Vacuum truck vent samples consistently exceeded our upper limit of detection for heterotrophic bacteria analysis, which varied between 5256 and 13,140 CFU m^−3^ depending on air sample volume (see SI for sample volume information). All actual levels reported in Table 1 could be markedly higher considering that shear during impingement in high flow samplers has been shown to significantly injure or kill bacteria (Terzieva et al., 1996). This comment is also valid for the quantification of *E. coli* reported in the next section. Although sampling was limited by the requirement to collect large volumes of air in a short time, these results suggest that bioaerosols of heterotrophic bacteria are generated during pit latrine emptying.

**Table 1:** Plating results for total coliforms and total heterotrophic bacteria of air samples. Values in parenthesis next to coliforms are *E. coli* counts, when there was a positive detection; no value in parenthesis means that there was no detection of *E. coli.*

	Total coliforms (*CFU m*^*−3*^)					Total heterotrophic bacteria (*CFU m*^*−3*^)			
Pit #	Background	Pit emptying	Vacuum truck vent	During fluidization^a^	After emptying	Background	Pit emptying	Vacuum truck vent	After emptying
1	2	4	–	790 (350)	–	442	600	>13,140^b^	–
2	1	10 (1)	17	–	–	>2628^b^	>13,140^b^	>5256^b^	–
3	12	–	–	–	–	–	–	–	–
4	3	5 (2)	–	–	3	384	782	–	322
5	2 (1)	7	–	–	3	695	1505	–	436
6	<1	–	<1	–	1	135	198	>5256^b^	254
7	14 (1)	–	–	–	–	>2628^b^	>5256^b^	>5256^b^	>2628^b^

(–): no sample was taken.

a Sample taken during fluidization step of pit emptying.

b Sample exceeded upper detection limit; upper limit is reported.

### 3.3 Quantification of *E.coli*, total coliforms

Total coliforms were detected in all of the air samples collected during active emptying and the majority of background samples (Table 1). The concentrations of total coliforms measured during pit emptying was approximately 2 to 3.5 times greater than concentrations measured in background samples (Table 1, Pits 1, 4, and 5) with one pit location having a 10 fold increase (Pit 2). *E. coli* was detected in two air samples taken during active emptying (Pits 2 and 4) and in two background samples (Pits 5 and 7), both at 2 CFU m^−3^ or less. The highest concentrations of airborne total coliforms (790 CFU m^−3^) and *E. coli* (350 CFU m^−3^) were detected during fluidization which was conducted at all pits, but only monitored at Pit 1. While the procedure was monitored only once, the results show the obvious potential for fluidization to create large volumes of concentrated bioaerosols. Personal protective equipment could limit the risk to the workers, but such large volumes of aerosols could pose much higher risk to the home owners or passersby than any of the other practice the emptiers used. These data show the potential for aerosolization of fecal indicator bacteria during specific sludge handling operations.

### 3.4 Molecular results

The Luminex xTAG GPP assay, which allows for the detection of 15 enteric pathogens, was used to assess the presence of select enteropathogens in air during pit emptying operations, as well as in the sludge of four of the pits. Of 42 individual air samples and 4 sludge samples collected, 38 had no pathogens detected, while eight were positive for enterotoxigenic *Escherichia coli* (ETEC) LT/ST (Table 2.). All eight samples testing positive for ETEC were air samples. No sludge samples tested positive for enteric pathogens, possibly a result of excessive dilution of the samples during processing coupled with the relatively high detection limits of the GPP assay (2.2 × 10^2^–3.75 × 10^6^ CFU or copies/mL stool according to the manufacturer). The GPP was designed for use with stool specimens and has not been optimized for other sample matrices, such as latrine waste, where target pathogen concentrations may be lower. Neither the culture-based methods nor the GPP allowed for quantification of concentration of ETEC in the air samples, though based on the air volume sampled, it was at least 1 to 10 CFU m^−3^.

**Table 2:** Summary of enteric pathogen detections.

Pit #	Air sample type and location	Enteric pathogen detected	Agar type	Detection limit (*CFU m*^*−3*^)
1	During fluidization	ETEC LT/ST	MI Agar	10
2	Truck vent	ETEC LT/ST	Nutrient Agar	2
	Truck vent	ETEC LT/ST	MI Agar	5
	During pit emptying	ETEC LT/ST	Nutrient Agar	5
5	During pit emptying	ETEC LT/ST	Nutrient Agar	1
	Background	ETEC LT/ST	Nutrient Agar	1
	Open air petri dish	ETEC LT/ST	Nutrient Agar	NA
7	Post-emptying, during cleanup	ETEC LT/ST	Nutrient Agar	1

ETEC was the only pathogen detected. ETEC is especially of note as being a major cause of diarrhea, and is responsible for around 400,000 children deaths per year (von Mentzer et al., 2014). Culture methods did not allow for detection or propagation of viruses or protozoa and therefore results of molecular analyses were biased in favor of detection of pathogenic bacteria in the coliform group. Nonetheless, the presence of an important human enteric pathogen such as ETEC in air samples is significant because airborne transport is not currently considered a significant route of exposure.

### 3.5 Implications of bioaerosols generated during pit emptying

To our knowledge, this is the first work to report on bioaerosols generated during pit latrine emptying, as well as to show the presence of at least one enteric pathogen (ETEC) in these bioaerosols. Sampling before, during and after pit emptying revealed an increase in total heterotrophic bacteria and total coliform bioaerosol concentration during pit emptying operations. Total coliforms and generic *E. coli* were generally present in lower concentrations than their heterotrophic counterparts. The process of sludge fluidization produced high concentrations of both total coliforms and *E. coli* and warrants further research as a potentially high-risk activity. Of 42 air samples tested for enteric pathogens, eight were positive for ETEC LT/ST, across four separate pit latrines. This exploratory work was limited by the size of the data set, and the methodology excluded detection of virus and protozoa, while being in part biased towards coliform bacteria. Nonetheless, the results indicate that pit emptying can generate bioaerosols, and more importantly, some of these bioaerosols contain known enteric pathogens. Currently, the primary pathway of exposure considered for enteric pathogens is the fecal-oral route (World Health Organization, 2004). However, this work shows that greater attention should be placed on the aeromicrobiological pathway, with special consideration for operations such as emptying pit latrines and vacuum trucks. Currently, the magnitude of the risk of the aeromicrobiological pathway relative to the oral route remains unknown. However, with greater understanding of the airborne exposure pathways and of the practices that result in bioaerosols during fecal sludge management, specific control methods, appropriate personal protective measures, and best operating practices can be developed and implemented.

## Appendix A Supplementary data

Supplementary material Image 1
