# Supplementary material for: Bioaerosol emissions associated with pit latrine emptying operations
Source: Sci Total Environ. 2019 Jan 15;648:1082–6. doi: 10.1016/j.scitotenv.2018.08.147 (PMC6234106; doi:10.1016/j.scitotenv.2018.08.147)

Supporting information to:

Bioaerosol Emissions Associated with Pit Latrine Emptying Operations

Stewart Farling, Tate Rogers, Jacqueline S. Knee, Elizabeth Tilley, Joe Brown, Marc A. Deshusses

Contact for correspondence: marc.deshusses@duke.edu

Table S1. Listing of all the field samples taken (continued on following pages)

Pit #	Sample	Sample Time	Sample Volume	Agar Type*	qPCR Sample^	Notes
#	-	min	m^3	-	-	
1	Flat	166	-	NA	Y	2 hours and 46 minutes out
	Flat	163	-	MI		2 hours and 43 minutes out
	Pit - Background	5	0.5	NA		Sampled before emptying
	Pit - Background	1	0.1	NA		Sampled before emptying
	Pit - Background	5	0.5	MI	Y	Sampled before emptying
	Downwind	10	1	NA	Y	
	Downwind	10	1	MI		
	Vent	0.5	0.05	NA		Vacuum Truck Blower Vent
	Vent	1	0.1	MI		Vacuum Truck Blower Vent
	Vent	2	0.2	NA	Y	Vacuum Truck Blower Vent
	Vent	2	0.5	MI		Vacuum Truck Blower Vent
	Pit	2	0.2	NA	Y	
	Fluidization	1	0.1	MI	Y	Sample in latrine adjacent to pit, latrines connected to same pit, aerosols visible in this stall

*NA = Nutrient Agar
*MI = MI Agar, selective for *E. coli* and Coliforms
^ = Y indicates sample was extracted for qPCR analysis

Pit #	Sample	Sample Time	Sample Volume	Agar Type*	qPCR Sample^	Notes
#	-	min	m^3	-	-	
2	Background	10	1	MI		
	Background	10	1	NA	Y	
	Flat	10	1	NA		
	Flat	10	1	MI		
	Pit	1	0.1	MI		
	Pit	10	1	MI	Y	
	Vent	5	0.5	MI		
	Vent	10	1	MI	Y	
	Downwind	10	1	MI	Y	
	Flat	193	-	MI		Downwind Location
	Pit	1	0.1	NA		
	Pit	2	0.2	NA	Y	
	Pit	5	0.5	NA	Y	
	Vent	0.5	0.05	NA		Vacuum Truck Blower Vent
	Vent	2	0.2	NA	Y	Vacuum Truck Blower Vent
	Vent	5	0.5	NA	Y	Vacuum Truck Blower Vent
	Downwind	10	1	NA	Y	
	Flat	193	-	NA	Y	Downwind Location
	Flat	193	-	NA	Y	Upwind Shower Location
	Plated-Sludge	-	-	NA	Y	
	Plated-Sludge	-	-	MI	Y	1 mL sludge sample diluted into 100 mL sterile water

Pit #	Sample	Sample Time	Sample Volume	Agar Type*	qPCR Sample^	Notes
#	-	min	m^3	-	-	
3	Background	10	1	NA	Y	
	Background	10	1	MI		
	Pit	10	1	MI		Pit emptying was halted 2 minutes after 10 minute sample was started, but allowed to finish
	Pit	5	0.5	NA	Y	
4	Background	10	1	MI		Samples taken after PIT 3 was emptied nearby
	Background	10	1	NA	Y	Samples taken after PIT 3 was emptied nearby
	Pit	10	1	MI		
	Pit	5	0.5	NA	Y	
	Pit-Post Clean	10	1	MI		Pit was emptied before full sampling could complete. Unplanned "post cleaning" samples added after workers completed cleaning the area
	Pit-Post Clean	5	0.5	NA		
	Pit-Post Clean	10	1	NA	Y	
5	Background	10	1	MI		
	Background	10	1	NA	Y	
	Pit	10	1	MI		
	Flat	79	-	MI		Pit location
	Pit-Post Clean	10	1	MI		
	Pit	1	0.1	NA	Y	
	Pit	2	0.2	NA		
	Pit	5	0.5	NA	Y	
	Pit	10	1	NA	Y	
	Flat	79		NA	Y	Pit location
	Flat	79		NA	Y	Window Seal
	Flat	79		NA	Y	Clothes Line Location
	Pit-Post Clean	10	1	NA		
	Sludge				Y	1 mL sludge sample diluted into 100 mL sterile water

Pit #	Sample	Sample Time	Sample Volume	Agar Type*	qPCR Sample^	Notes
#	-	min	m^3	-	-	
6	Background	10	1	MI		
	Background	10	1	NA	Y	
	Flat	50		MI		Pit location
	Flat	50		MI		Window Seal
	Vent	10	1	MI		Vacuum Truck Blower Vent
	Pit-Post Clean	10	1	MI		
	Pit	5	0.5	NA	Y	
	Flat	50		NA	Y	Pit location
	Vent	5	0.5	NA	Y	Vacuum Truck Blower Vent
	Pit-Post Clean	5	0.5	NA	Y	
	Sludge				Y	1 mL sludge sample diluted into 100 mL sterile water
7	Background	10	1	NA	Y	
	Background	10	1	MI		
	Pit	5	0.5	NA	Y	Sampler battery was dying, prioritized NA samples for qPCR
	Vent	5	0.5	NA	Y	Sampler battery was dying, prioritized NA samples for qPCR
	Pit-Cleaning	10	0.5	NA	Y	Sampler battery was dying, prioritized NA samples for qPCR
	Flat	148	-	NA	Y	Pit location
	Flat	148	-	NA	Y	Pit location
	Sludge				Y	1 mL sludge sample diluted into 100 mL sterile water
Sterile Blanks	Lab Control	10	1	NA	Y	10 minute bioaerosol sample of lab space used to prepare agar plates
	Blank			MI		Agar prep sterilization blanks - Batch 1
	Blank			NA		Agar prep sterilization blanks - Batch 1
	Blank			MI		Agar prep sterilization blanks - Batch 2
	Blank			NA		Agar prep sterilization blanks - Batch 2
	Blank			MI		Agar prep sterilization blanks - Batch 3
	Blank			NA		Agar prep sterilization blanks - Batch 3

*NA = Nutrient Agar

*MI = MI Agar, selective for *E. coli* and Coliforms

^ = Y indicates sample was extracted for qPCR analysis

Pit 7 Sampling Layout

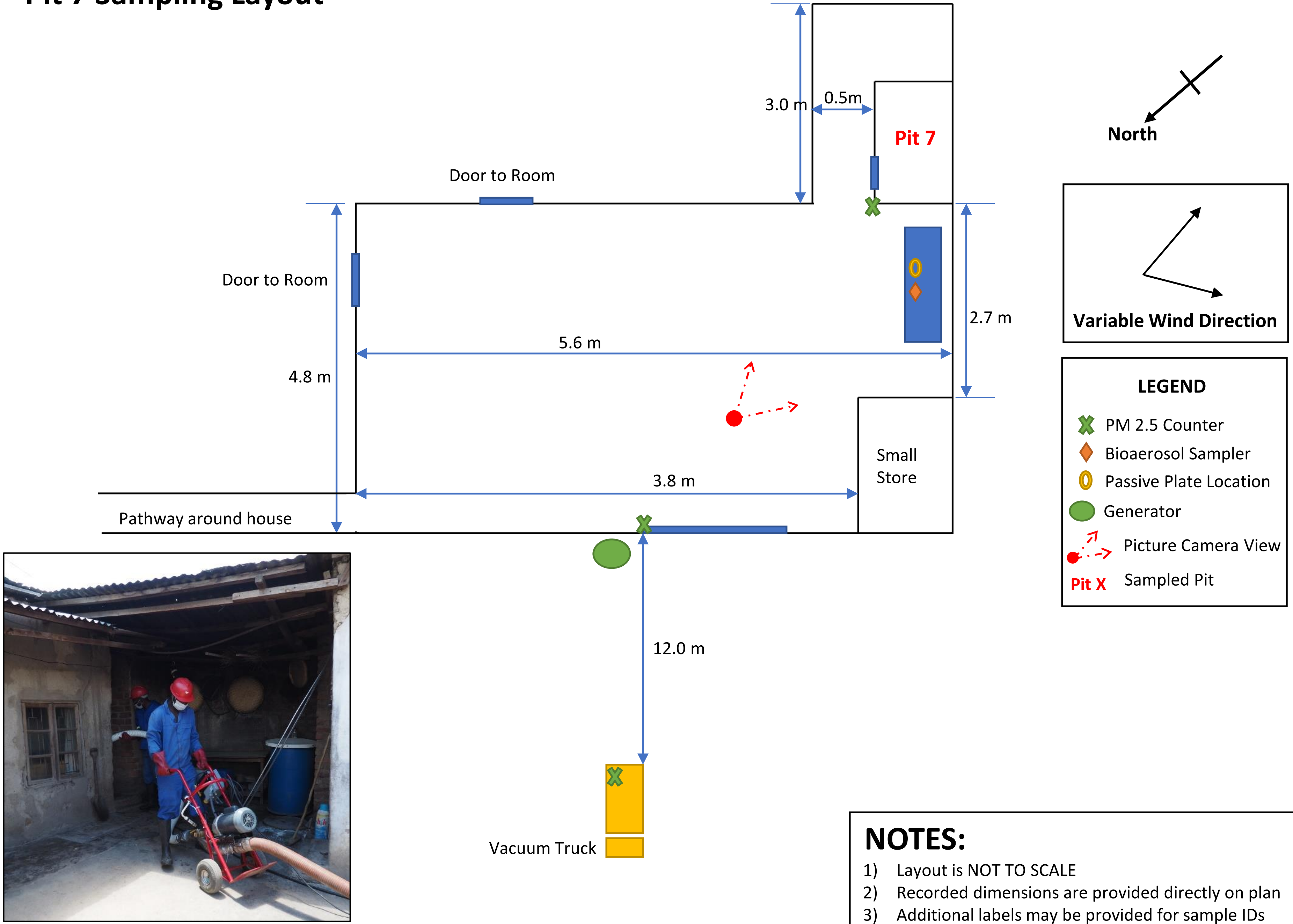

NOTES:

1) Layout is NOT TO SCALE
2) Recorded dimensions are provided directly on plan
3) Additional labels may be provided for sample IDs

Pit 6 Sampling Layout

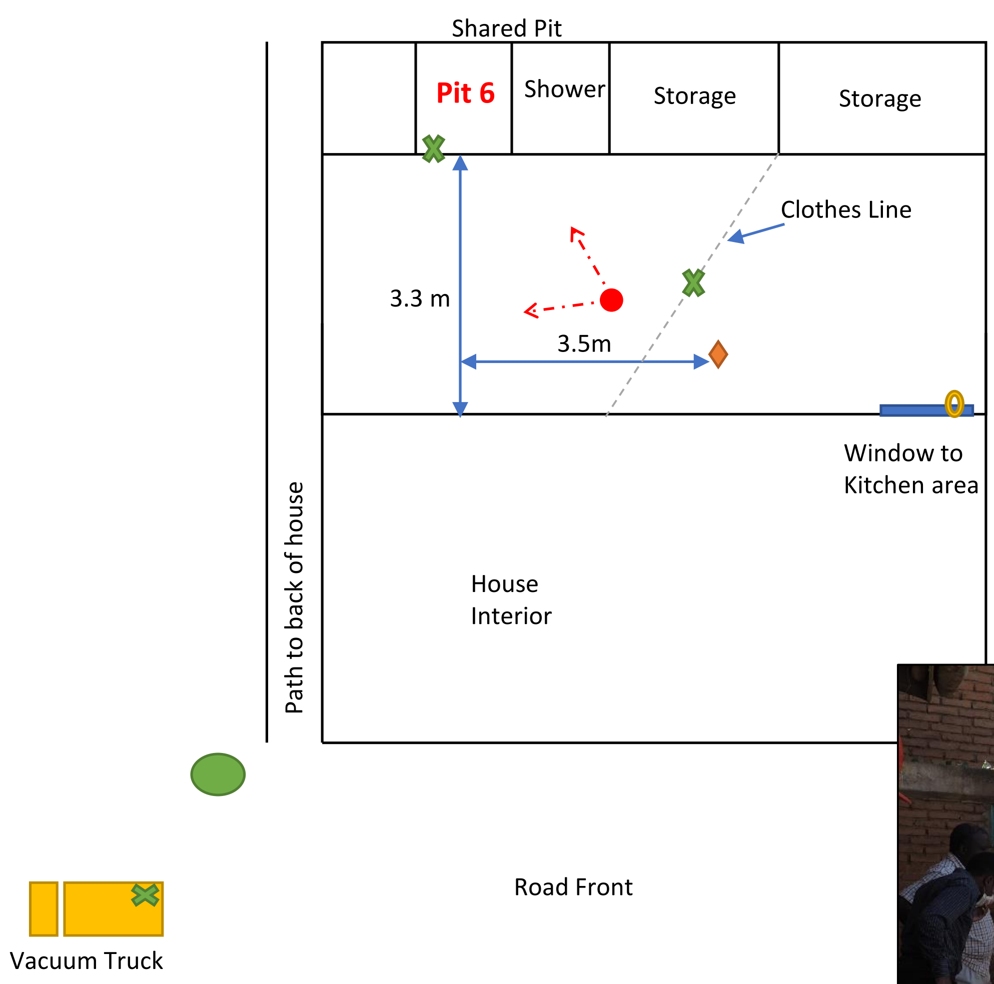

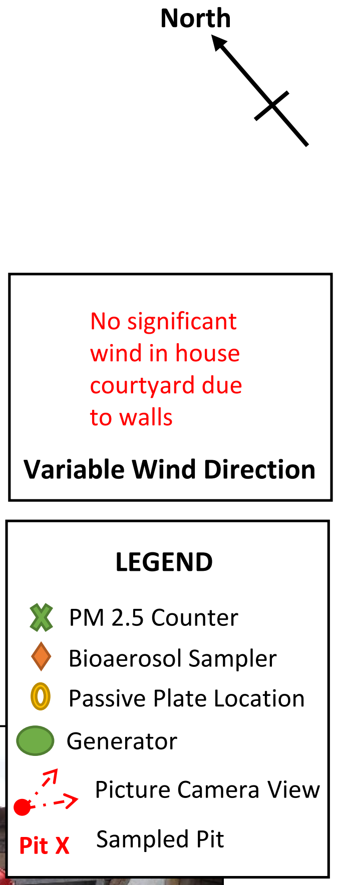

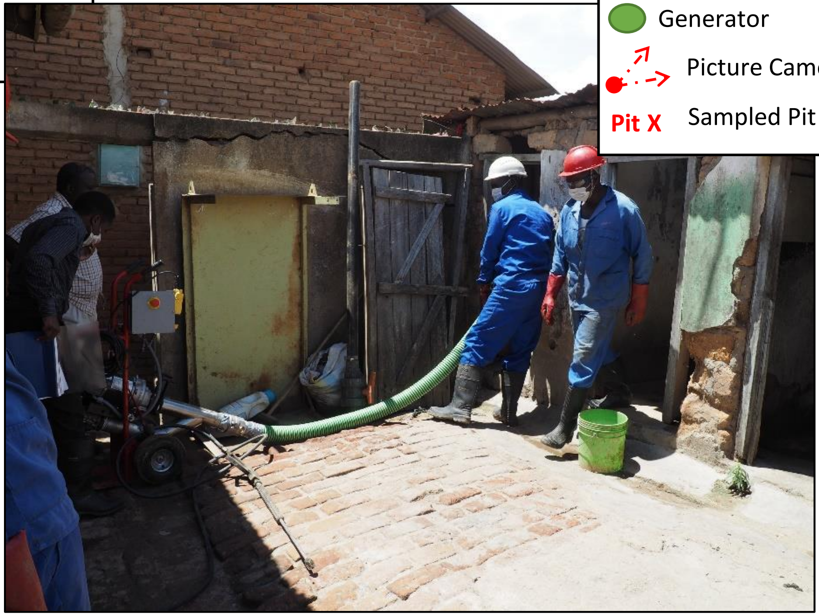

NOTES:

1) Layout is NOT TO SCALE
2) Recorded dimensions are provided directly on plan
3) Additional labels may be provided for sample IDs

Pit 5 Sampling Layout

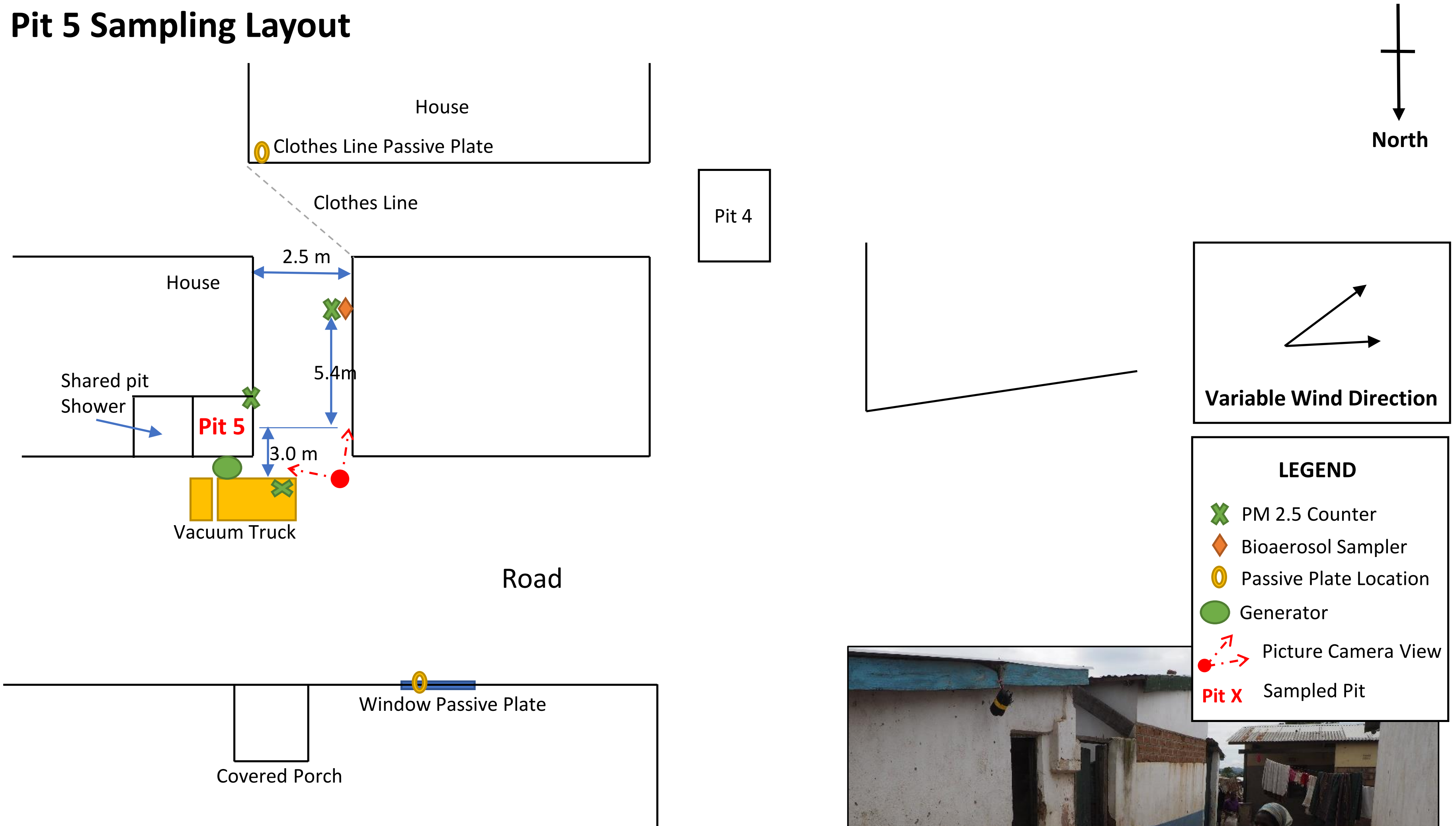

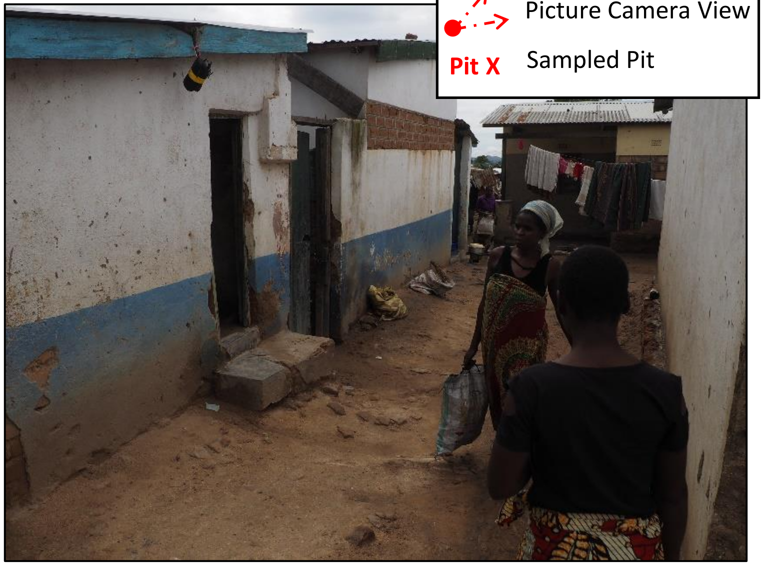

NOTES:

1) Layout is NOT TO SCALE
2) Recorded dimensions are provided directly on plan
3) Additional labels may be provided for sample IDs

Pit 4 Sampling Layout

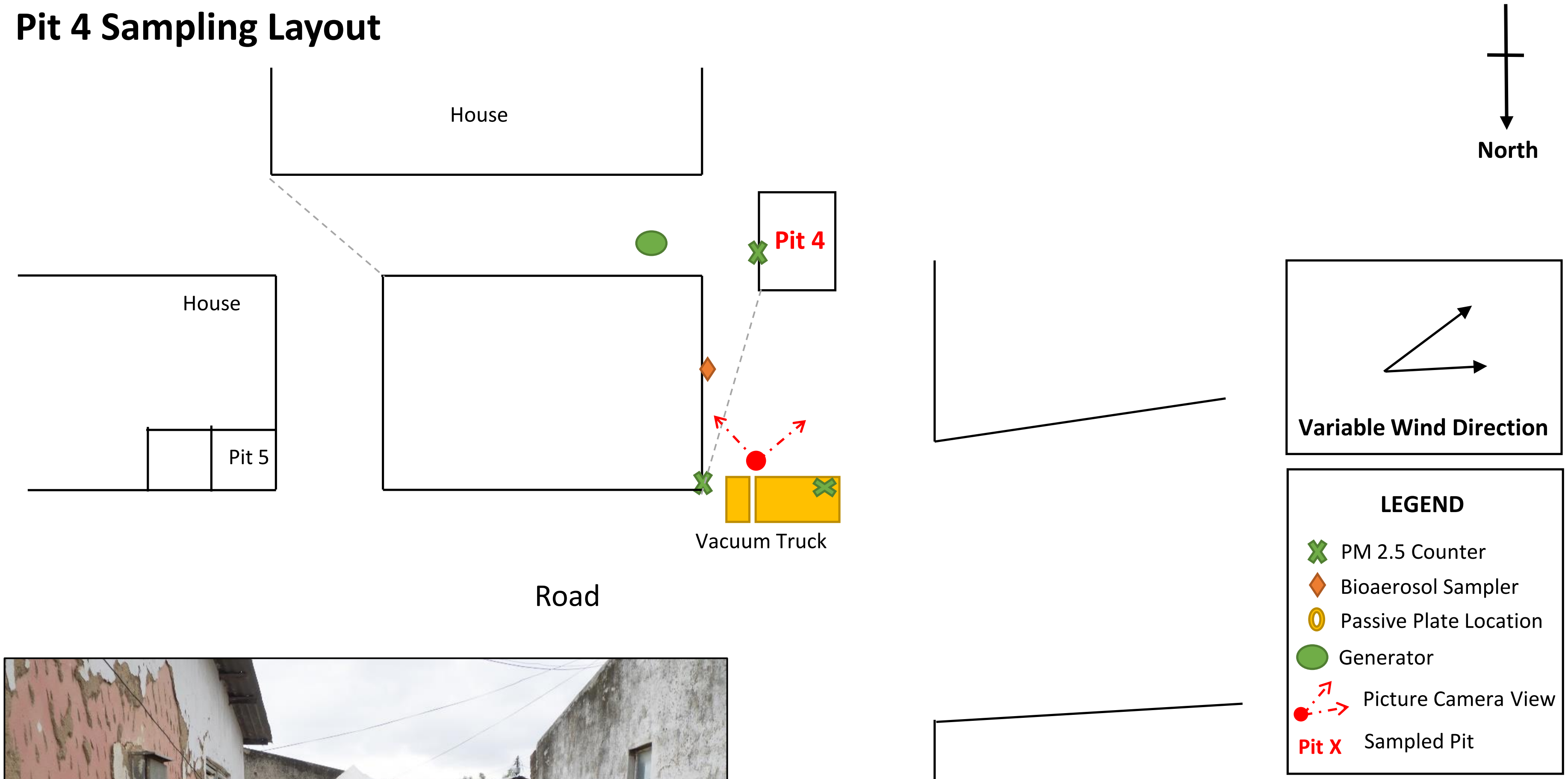

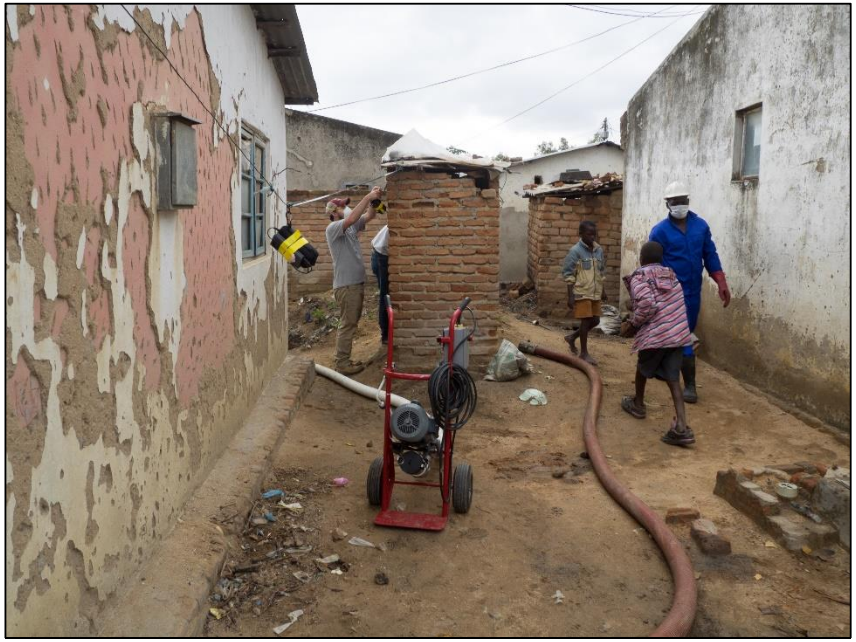

NOTES:

1) Layout is NOT TO SCALE
2) Recorded dimensions are provided directly on plan
3) Additional labels may be provided for sample IDs
4) Pit 3 and 4 were sampled the same day

Pit 3 Sampling Layout

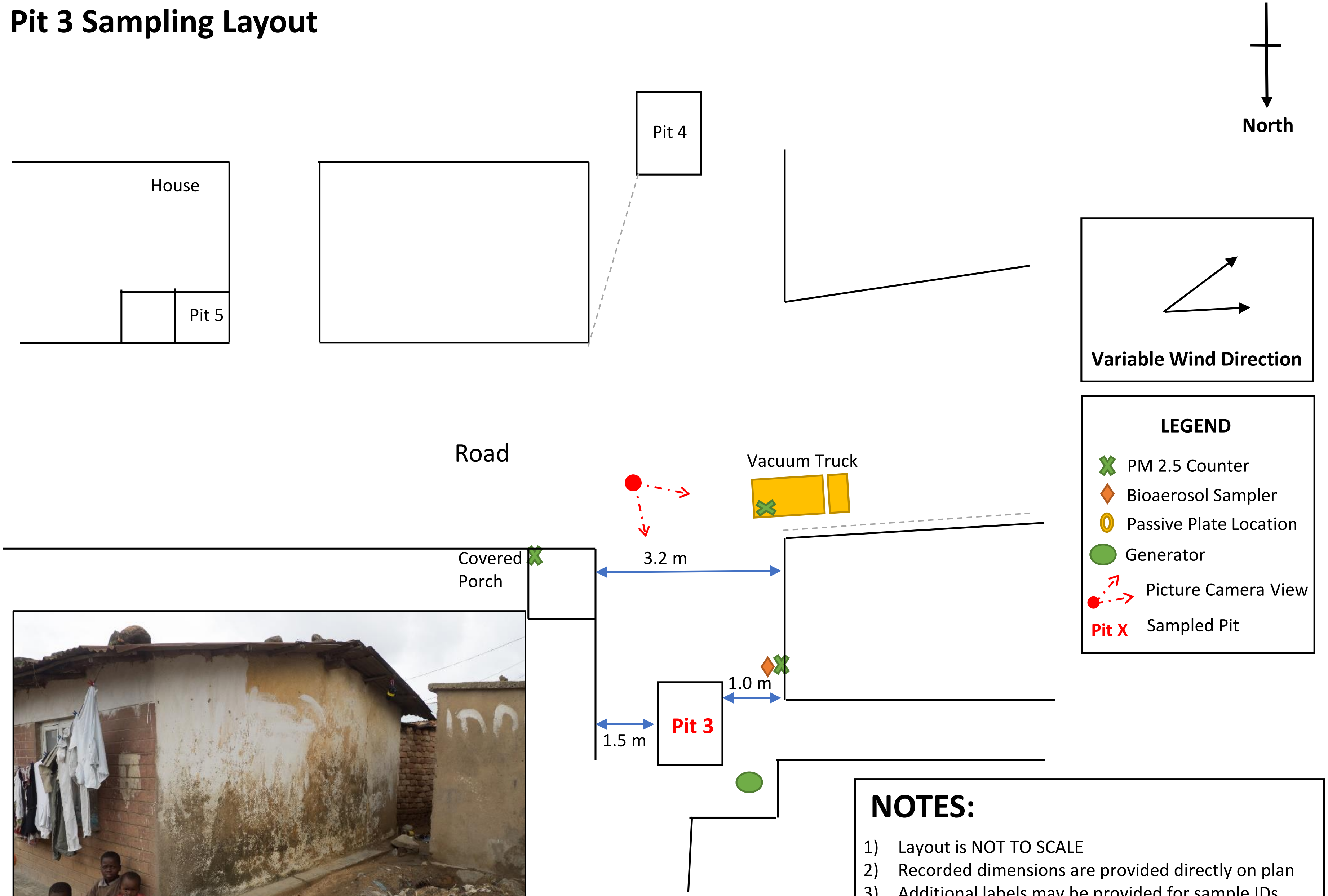

NOTES:

1) Layout is NOT TO SCALE
2) Recorded dimensions are provided directly on plan
3) Additional labels may be provided for sample IDs
4) Pit 3 and 4 were sampled the same day

Pit 2 Sampling Layout

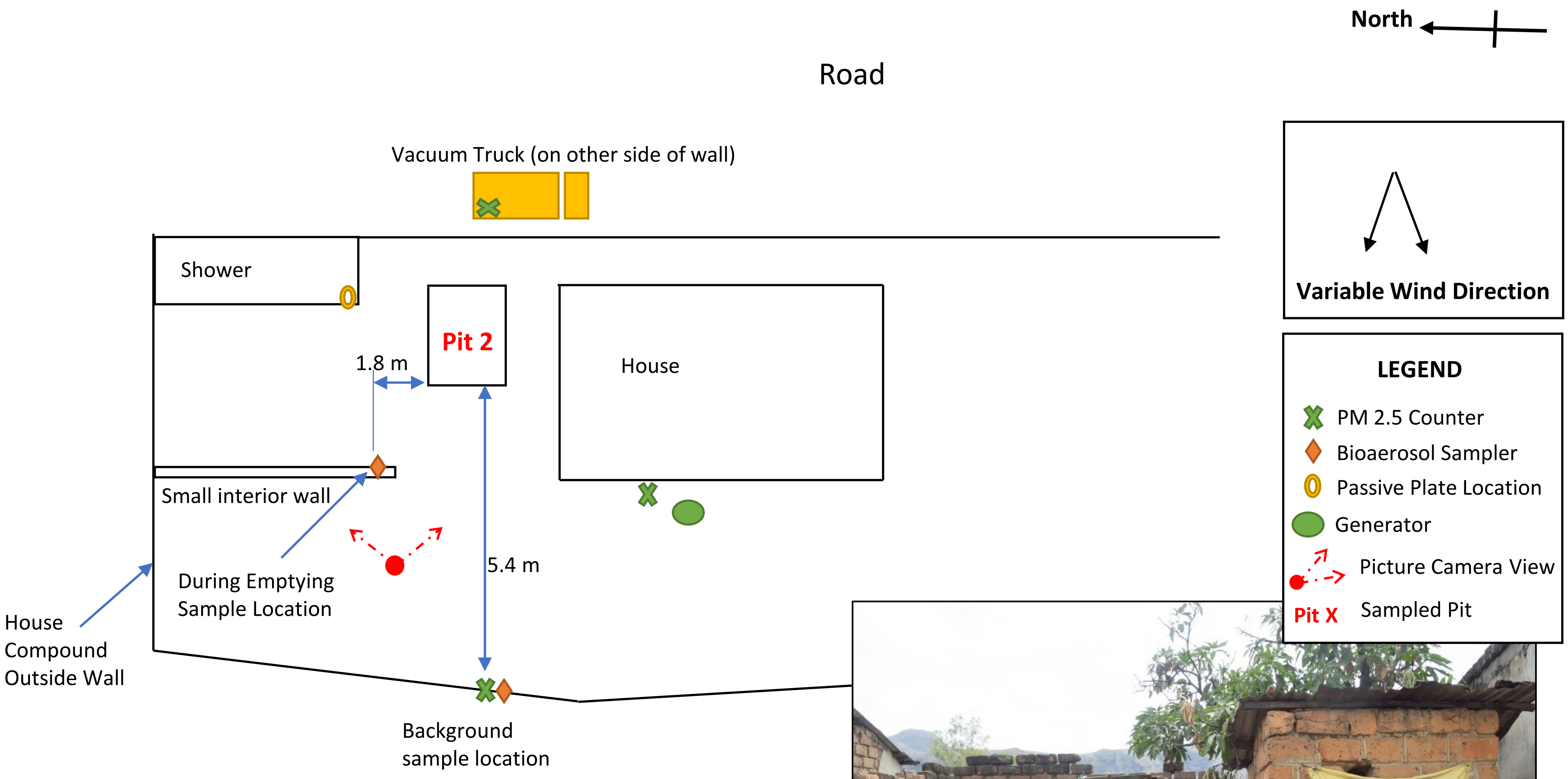

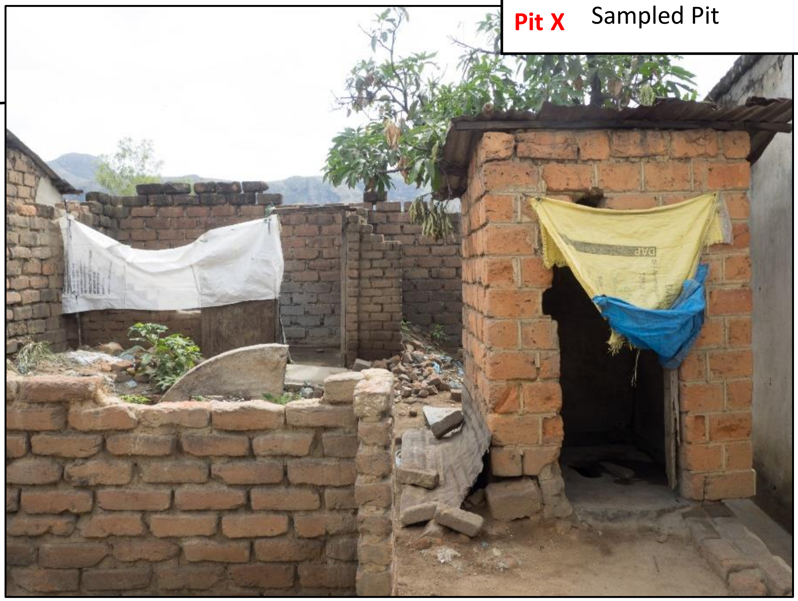

NOTES:

1) Layout is NOT TO SCALE
2) Recorded dimensions are provided directly on plan
3) Additional labels may be provided for sample IDs

Pit 1 Sampling Layout

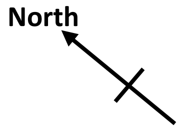

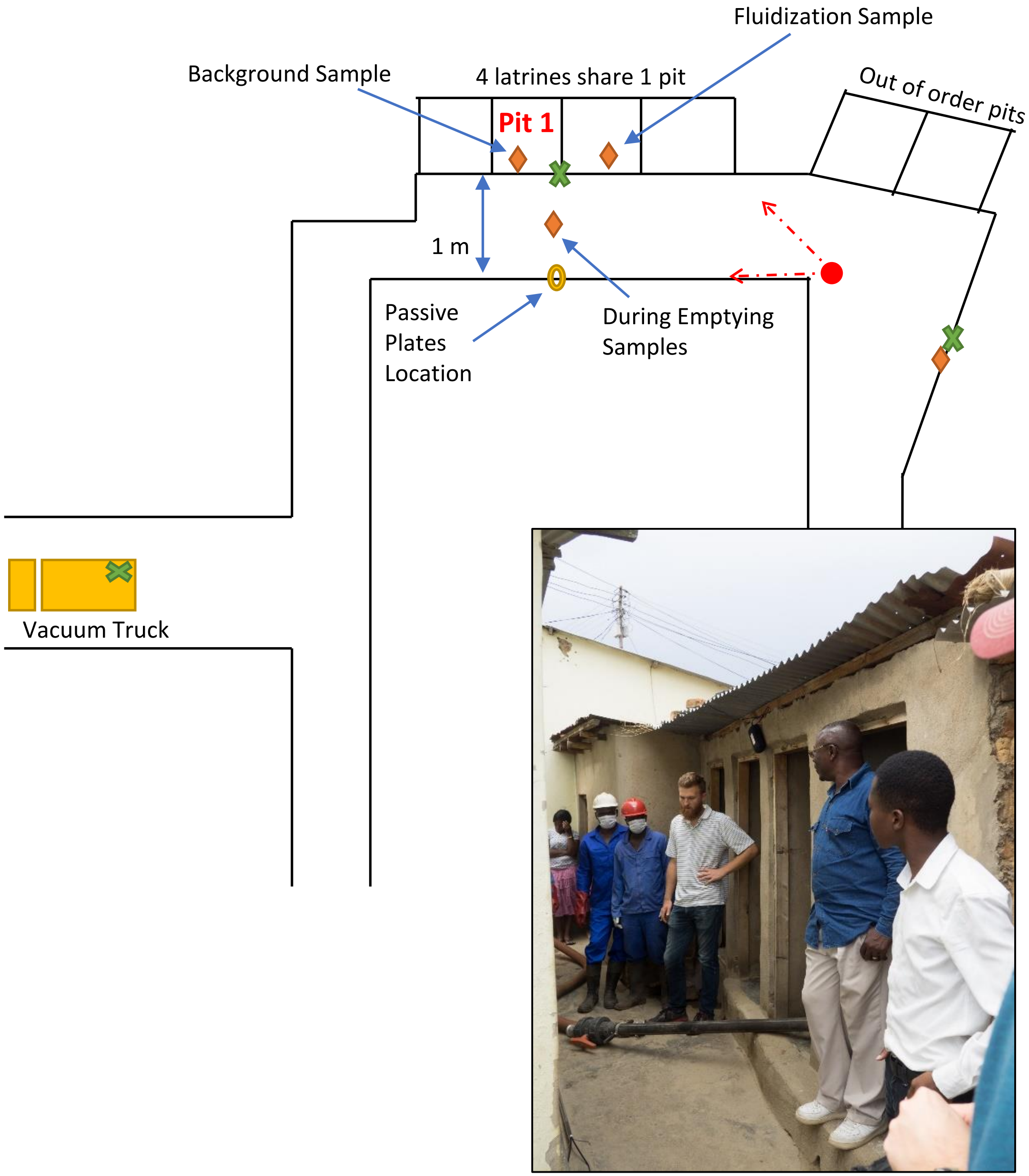

No significant wind due to walls

Variable Wind Direction

LEGEND

- PM 2.5 Counter
- Bioaerosol Sampler
- Passive Plate Location
- Generator
- Picture Camera View
- Pit X — Sampled Pit

NOTES:

1) Layout is NOT TO SCALE
2) Recorded dimensions are provided directly on plan
3) Additional labels may be provided for sample IDs

- Pit 7 other angles

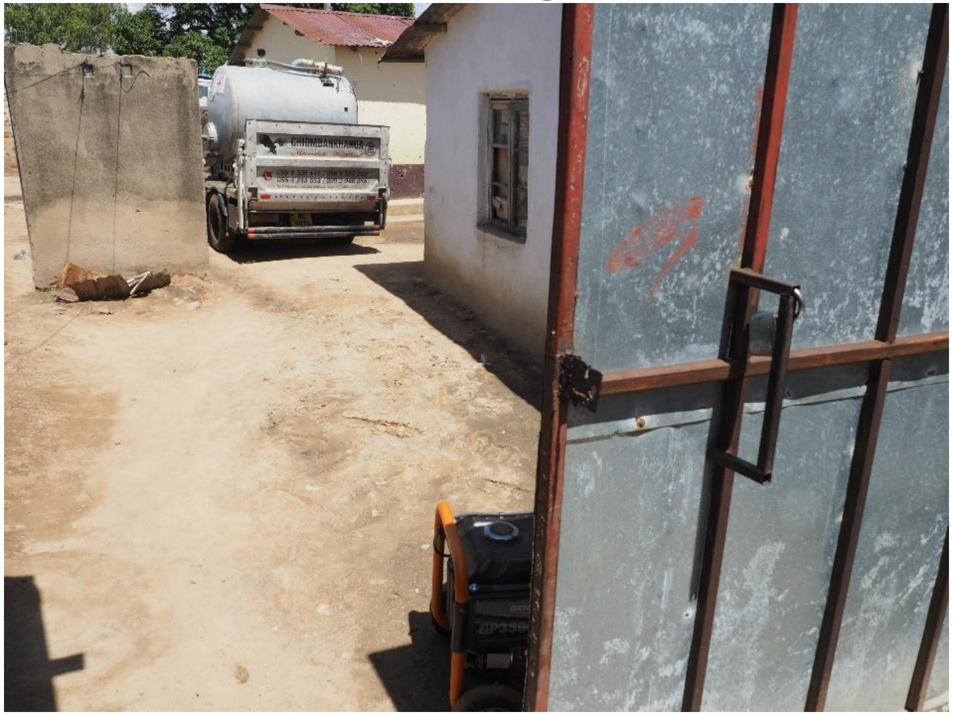

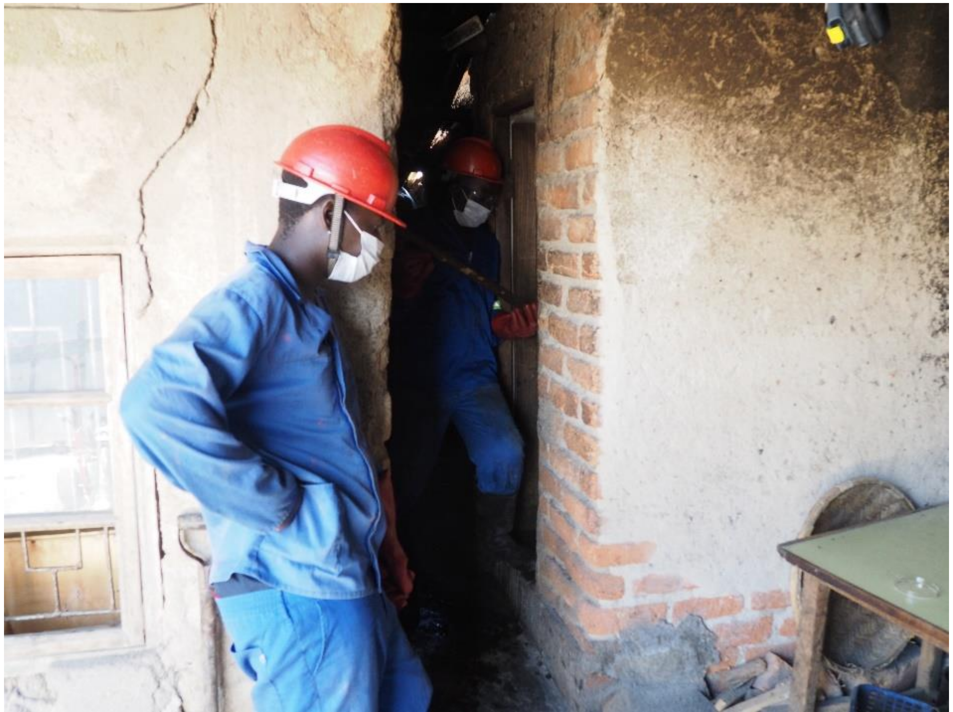

Supplement: Supplementary file 1 — Supplementary material [file mmc1.pdf]
